# Evolution and driving factors of inequality in CO_2_ emissions from agricultural energy consumption in China

**DOI:** 10.1038/s41598-024-63977-x

**Published:** 2024-06-06

**Authors:** Xiaojing Zhao, Xuke Li, Yanling Xi

**Affiliations:** 1 School of Business (MBA Education Center), Henan University of Science and Technology, 263 Kaiyuan Avenue, Luoyang, 471023 China; 2 Institute of Ecological Civilization, Tianjin Academy of Social Sciences, Tianjin, 300191 China

**Keywords:** Inequality, Kaya–Theil model, Kernel density estimation, Energy intensity, CO_2_ emission intensity, Agricultural economic development, Environmental economics, Environmental impact

## Abstract

The inequality in CO_2_ emissions from agricultural energy consumption is a major challenge for coordinating low-carbon agricultural development across regions in China. However, the evolutionary characteristics and driving factors of inequality in China’s agricultural energy-related CO_2_ emissions are poorly understood. In response, the Kaya–Theil model was adopted to examine the three potential factors influencing CO_2_ emission inequality in China’s agricultural energy consumption. The results revealed that, from 1997 to 2021, agricultural energy-related CO_2_ emissions per capita showed a significant upward trend, with prominent polarization and right-tailing phenomena. Overall, the inequality was on a downward trend, with the Theil index falling from 0.4109 in 1997 to 0.1957 in 2021. Meanwhile, the decomposition of the national inequality revealed that the within-group inequality declined from 0.3991 to 0.1634, which was greater than between-group inequality, based on zoning the 28 provinces into three grain production functional areas. As for the three kaya factors, the energy intensity contributed the most to the overall inequality, followed by the agricultural economic development and CO_2_ emission intensity. Based on these results, this study provided some potential strategies to reduce agricultural-related CO_2_ emissions.

## Introduction

Agriculture is a pivotal player in the global climate change^1^. China is currently the world’s largest producer of agricultural products^2^. The growth in agricultural production can be attributed to the increased investments and inputs^3–5^, but it has also led to higher reliance on energy, resulting in an annual growth rate of 2% in agricultural energy consumption^6–10^. Accompanied by a further increase in energy consumption, energy-related CO_2_ emissions in China’s agricultural sector were becoming an issue that cannot be underestimated^11,12^. In 2020, agricultural energy-driven CO_2_ emissions reached 217.67 million tons, accounting for 32.88% of the country’s total agricultural carbon emissions^13^. Notably, it was estimated that agricultural energy-driven CO_2_ emissions were still growing at an annual rate of 2.40%^14^. Agricultural carbon emission reduction is imperative in addressing the challenges posed by the greenhouse effect^15^. While crop planting and livestock husbandry are the main sources of agricultural carbon emissions^16^, the impact of agricultural energy consumption on CO_2_ emissions, the most uncertain factor in reaching emissions peak, should not be overlooked^11,17^.

Agricultural energy conservation and carbon emission reduction are crucial for the successful implementation of China’s Dual-Carbon strategy^18^. However, due to China’s vast territory and significant differences in agricultural resources, production conditions, industrial structure and energy demands, there is also notable inequality in agricultural energy-related CO_2_ emissions across regions^19,20^. Among all the provinces in China, Qinghai had the lowest percentage of agricultural energy-related CO_2_ emissions in its total agricultural CO_2_ emissions, accounting for only 2.76%. In contrast, Tianjin had the highest percentage of 41.99%^16^. Regarding CO_2_ emissions from agricultural energy consumption in China from 1997 to 2016, the province ranked first emitted 43.29 times more than the last-ranked province^21^. Compared with 2012, the use of petroleum products and electricity in agriculture was estimated to increase by 110.6% and 139.1% by 2030^6^. Thus, from the perspectives of emission structure, emission amount and growth rate of energy consumption, agricultural energy-related CO_2_ emissions exhibited significant inequality.

One of the most crucial issues in sharing the responsibility for reducing CO_2_ emissions is the unequal distribution of CO_2_ emissions among different regions. Furthermore, with the recent launch of the agricultural modernization strategy towards 2035^22^, the inequality in agricultural energy-related CO_2_ emissions is set to rise unless actions are taken^23^. The CO_2_ emission inequality is a crucial obstacle to achieving collective horizontal reduction of emissions among various grain production functional areas. Despite this pressing issue, the evolution characteristics and driving factors of CO_2_ emission inequality in China’s agricultural energy consumption are still unclear. A one-size-fits-all carbon emission reduction policy is not suitable for China. Therefore, a comprehensive study on this topic is necessary, which can be conducive to develop tailored policies to achieve more equitable CO_2_ emission reduction in China.

## Literature review

Designing an inter-regional responsibility sharing mechanism for climate change mitigation requires the knowledge of carbon emission distributions^24^. However, all humans contribute to climate change but not equally^25,26^. Therefore, it is vital to assess the CO_2_ emission inequality to ensure equitable access to CO_2_ emissions rights for all humans^27^. As early as 1996, the IPCC evaluated the inequality in CO_2_ emissions at an international level^28^. Since then, measuring CO_2_ emission inequality has become a topic of widespread concern^29–32^. The Gini coefficient, the Atkinson index and the Theil index are the most commonly used measures of inequality^33–39^.

In 1997, Heil and Wodon introduced the Gini coefficient and investigated the contributions of both poor and rich countries to global CO_2_ emission inequality^40^. However, researchers found that when applying the Gini coefficient, it produced cross-terms after decomposition that was difficult to explain^41–45^. In contrast, the Theil index, which satisfies the principles of additivity and decomposability, is known to be better suited for exploring the group contribution and factors driving the CO_2_ emission inequality^46,47^. Additionally, the Atkinson index has a monotonic correspondence with the Theil index and can be converted to eliminate any residual^48^. Therefore, the Theil index is preferred for evaluating the CO_2_ emission inequality over both the Atkinson index and the Gini coefficient^49^.

Per capita CO_2_ emissions are considered to be the relatively best choice to measure CO_2_ emission inequality^24^. This is because per capita CO_2_ emissions reflect the idea of interpersonal equity by taking into account population size^50,51^. In other words, everyone has equal rights to carbon emissions^52^. Based on the eight regions identified by multi-regional input–output model in China, Hu and Wang used the Theil index decomposition technique to assess the regional differences of agricultural energy-related CO_2_ emissions, and showed that the between-group difference contributed greater to the total difference than within-group difference^23^. A study by Duro and Padilla analyzed the per capita CO_2_ emissions of 114 countries and decomposed the Theil index into three kaya factors. The results revealed that affluence was the main contributor to per capita CO_2_ emission inequality, followed by CO_2_ emission intensity and energy intensity^53^. The Kaya-Theil approach has been widely used by researchers to explore the drivers of CO_2_ emission inequality from global, national, and sectoral perspectives^29,54–56^.

Given China’s significant role in global CO_2_ emission reduction, issues related to CO_2_ emissions have been examined from various perspectives^44^. However, the research on China's CO_2_ emission inequality is insufficient. Researchers have utilized the Kaya-Theil approach to examine this issue and have found that energy intensity contributed the most to CO_2_ emission inequality at the provincial level in China^42^. Using the same method, researchers have also investigated the factors influencing the CO_2_ emission inequality in different industries, such as the industry and construction sectors, as well as the inequality in CO_2_ emissions driven by different energy sources such as coal, oil, and natural gas^32,57–60^. In addition, the Kaya–Theil model has been applied to assess the CO_2_ emission inequality in China’s urban and rural residential sectors. The findings revealed that per capita disposable income was the primary factor affecting the inequality, followed by energy intensity and CO_2_ emission intensity, whereas the influence of consumption propensity could be ignored^61^.

It is crucial to have an overview of China’s CO_2_ emission inequality^26,51^. The existing literature on CO_2_ emission inequality has been conducted from the perspectives of geographical scale, economic sectors and energy sources. To the best of our knowledge, few studies have concerned inequality in China’s agricultural energy-related CO_2_ emissions. Nonetheless, to understand in detail of inequality in CO_2_ emissions from agricultural energy consumption is the key to formulating fair emission reduction strategies. Therefore, this study supplemented the existing literature by constructing a comprehensive analytical framework incorporating the Kaya-Theil model and Kernel density estimation. By doing so, this research identified the evolution of the inequality and brought about new insights to help understand the factors driving the inequality.

## Methods and data

### Kaya–Theil model

According to the Theil index^62^, a population-weighted inequality index, the inequality in CO_2_ emissions per capita from agricultural energy consumption can be described as follows:

1 $$T(c,p) = \sum\limits_{i} {p_{i} \ln \left( {\frac{{\overline{c} }}{{c_{i} }}} \right)} .$$

$p_{i}$ refers to the proportion of agricultural labor in the province $i$, $c_{i}$ indicates CO_2_ emissions per capita from agricultural energy consumption in the province $i$, $\overline{c}$ denotes the national average.

According to the Kaya identity^63^, the CO_2_ emissions per capita from agricultural energy consumption can be decomposed into the product of three factors:

2 $$\frac{{CO_{2,i} }}{{P_{i} }} = \frac{{CO_{2,i} }}{{E_{i} }} \times \frac{{E_{i} }}{{Q_{i} }} \times \frac{{Q_{i} }}{{P_{i} }} = a_{i} \times b_{i} \times y_{i} ,$$

where $c_{i} = a_{i} \cdot b_{i} \cdot y_{i} \cdot CO_{2,i}$ represents CO_2_ emissions due to agricultural energy consumption in province $i$, $E_{i}$ is the agricultural energy consumption, $Q_{i}$ is the agricultural value added, $P_{i}$ is the population engaged in agriculture in province $i$. Therefore, $a_{i}$, $b_{i}$, and $y_{i}$ refer to the CO_2_ emission intensity (CO_2_ emissions per unit of energy consumed), energy intensity, and the agricultural value added per capita.

According to Duro ^64^, the following three vectors are constructed:

3 $$c_{i}^{a} = a_{i} \cdot \overline{b} \cdot \overline{y} ,c_{i}^{b} = \overline{a} \cdot b_{i} \cdot \overline{y} ,c_{i}^{y} = \overline{a} \cdot \overline{b} \cdot y_{i} ,$$

where $\overline{a}$, $\overline{b}$, and $\overline{y}$ are the national averages. Then, the inequality of the vectors can be measured by the following Theil indexes:

4 $$T^{w} = \sum\limits_{i} {p_{i} \ln \left( {\frac{{\overline{c}^{w} }}{{c_{i}^{w} }}} \right)} ,\;{\text{where}}\;\overline{c}^{w} = \sum\limits_{i} {p_{i} c_{i}^{w} } ,w = a,b,y.$$

Following Duro and Padilla ^53^, the two terms, $\ln \left( {\frac{{\overline{c} }}{{\overline{c}^{a} }}} \right)$ and $\ln \left( {\frac{{\overline{c} }}{{\overline{c}^{b} }}} \right)$, are added together with the above Theil indexes.

5 $$T^{a} + \ln \left( {\frac{{\overline{c} }}{{\overline{c}^{a} }}} \right) = \sum\limits_{i} {p_{i} } \ln \left( {\frac{{\overline{a} }}{{a_{i} }}} \right),$$

6 $$T^{b} + \ln \left( {\frac{{\overline{c} }}{{\overline{c}^{b} }}} \right) = \sum\limits_{i} {p_{i} } \ln \left( {\frac{{\overline{b} }}{{b_{i} }}} \right),$$

7 $$T^{y} = \sum\limits_{i} {p_{i} } \ln \left( {\frac{{\overline{c}^{y} }}{{c_{i}^{y} }}} \right) = \sum\limits_{i} {p_{i} } \ln \left( {\frac{{\sum\limits_{i} {p_{i} \overline{a} \cdot \overline{b} \cdot y_{i} } }}{{\overline{a} \cdot \overline{b} \cdot y_{i} }}} \right) = \sum\limits_{i} {p_{i} } \ln \left( {\frac{{\overline{y} }}{{y_{i} }}} \right).$$

Finally, the formula (1) can be expressed as the sum of the three Theil indexes and the two terms.

8 $$\begin{aligned} &T^{a} + \ln \left( {\frac{{\overline{c} }}{{\overline{c}^{a} }}} \right) + T^{b} + \ln \left( {\frac{{\overline{c} }}{{\overline{c}^{b} }}} \right) + T^{y} \hfill \\& \quad = \sum\limits_{i} {p_{i} } \ln \left( {\frac{{\overline{a} }}{{a_{i} }}} \right) + \sum\limits_{i} {p_{i} } \ln \left( {\frac{{\overline{b} }}{{b_{i} }}} \right) + \sum\limits_{i} {p_{i} } \ln \left( {\frac{{\overline{y} }}{{y_{i} }}} \right). \hfill \\ & \quad = \sum\limits_{i} {p_{i} } \ln \left( {\frac{{\overline{a} }}{{a_{i} }} \cdot \frac{{\overline{b} }}{{b_{i} }} \cdot \frac{{\overline{y} }}{{y_{i} }}} \right) = \sum\limits_{i} {p_{i} \ln \left( {\frac{{\overline{c} }}{{c_{i} }}} \right)} = T\left( {c,p} \right) \hfill \\ \end{aligned}$$

Using the Shorrock methodology^65^, the overall inequality is decomposed into effects concerning the three factors identified.

1. Agricultural energy structure effect
  9 $$T^{A} = T^{a} + \frac{1}{2}\ln \left( {\frac{{\overline{c} }}{{\overline{c}^{a} }}} \right) = T^{a} + \frac{1}{2}\ln \left( {1 + \frac{{\sigma_{a,by} }}{{\overline{c}^{a} }}} \right).$$
2. Agricultural energy intensity effect
  10 $$T^{B} = T^{b} + \frac{1}{4}\ln \left( {1 + \frac{{\sigma_{a,by} }}{{\overline{c}^{a} }}} \right) + \frac{1}{2}\ln \left( {1 + \frac{{\overline{a} \times \sigma_{b,y} }}{{\overline{c}^{b} }}} \right).$$
3. Agricultural economic development effect
  11 $$T^{Y} = T^{y} + \frac{1}{4}\ln \left( {1 + \frac{{\sigma_{a,by} }}{{\overline{c}^{a} }}} \right) + \frac{1}{2}\ln \left( {1 + \frac{{\overline{a} \times \sigma_{b,y} }}{{\overline{c}^{b} }}} \right).$$

In addition, the overall inequality is decomposed into within-group inequality and between-group inequality based on three grain production functional areas.

12 $$T\left( {c,p} \right) = \sum\limits_{k = 1}^{K} {p_{k} T\left( {c,p} \right)_{k} } + \sum\limits_{k = 1}^{K} {p_{k} \ln \left( {\frac{{\overline{c} }}{{c_{k} }}} \right)} ,$$

where $p_{k}$ denotes the proportion of agricultural labor in the region $k$ of China, $T\left( {c,p} \right)_{k}$ indicates inequality in agricultural energy-related CO_2_ emissions per capita in region $k$, $c_{k}$ refers to agricultural energy-related CO_2_ emissions per capita in the region $k$.

### Kernel density estimation

The kernel density estimation is an important nonparametric method used to analyze various properties of a variable^66–68^. According to the previous literature^16,69^, this study utilized the Gaussian kernel function to reveal the evolution characteristics of CO_2_ emissions per capita from agricultural energy consumption, including distribution position, shape, ductility, and polarization phenomenon.

The density function of the variable $x$ is presented below:

13 $$f\left( x \right) = \frac{1}{nh}\sum\limits_{i = 1}^{n} {k\left( {\frac{{x_{i} - \overline{x} }}{h}} \right)} ,$$

where $k\left( \cdot \right)$ is the kernel function, $n$ is the number of values, $\overline{x}$ indicates the average of $x_{i}$. The bandwidth $h$ determines the accuracy of kernel density estimation and the smoothness of the kernel density curve. Generally, $h$ is the function of $n$, and the optimal solution is shown as follows:

14 $$h = \left( {4/3n} \right)^{\frac{1}{5}} \approx 1.06n^{{ - \frac{1}{5}}} .$$

### Data sources

At the beginning of the twenty-first century, China implemented a reform of its grain circulation system. This involved grouping the 31 provinces, autonomous regions, and municipalities into three distinct grain production functional areas: major grain-producing area, main grain-selling area, and grain balanced area. These areas were determined based on differences in the agricultural resource conditions and historical traditions of grain production and consumption.

In this study, major grain-producing area, major grain-selling area and grain balanced area were selected for research. As China does not have an official agricultural CO_2_ emission accounting system, the agricultural energy-related CO_2_ emissions from 1997 to 2021 were collected from the Carbon Emission Accounts & Datasets (CEADs). Following the IPCC sectoral approach^70^, the CEADs updated China’s carbon emission coefficient based on apparent energy consumption data and calculated the agricultural energy-related CO_2_ emissions of 30 provinces in mainland China (excluding Xizang due to unavailable data).

For agricultural energy consumption, the CEADs considered 16 types of fossil fuels consumed in agriculture. Data concerning agricultural energy consumption were acquired from the China Energy Statistical Yearbook. Hainan and Ningxia were not included in this study due to incomplete data. The data on agricultural value added and the population engaged in agriculture were acquired from the China Statistical Yearbook and China Agriculture Statistical Report. Among them, the data of agricultural value added were conversed with reference to the 1997 constant price.

## Results and discussion

### Distribution patterns of agricultural energy-related CO_2_ emissions per capita

**Figure 1:**
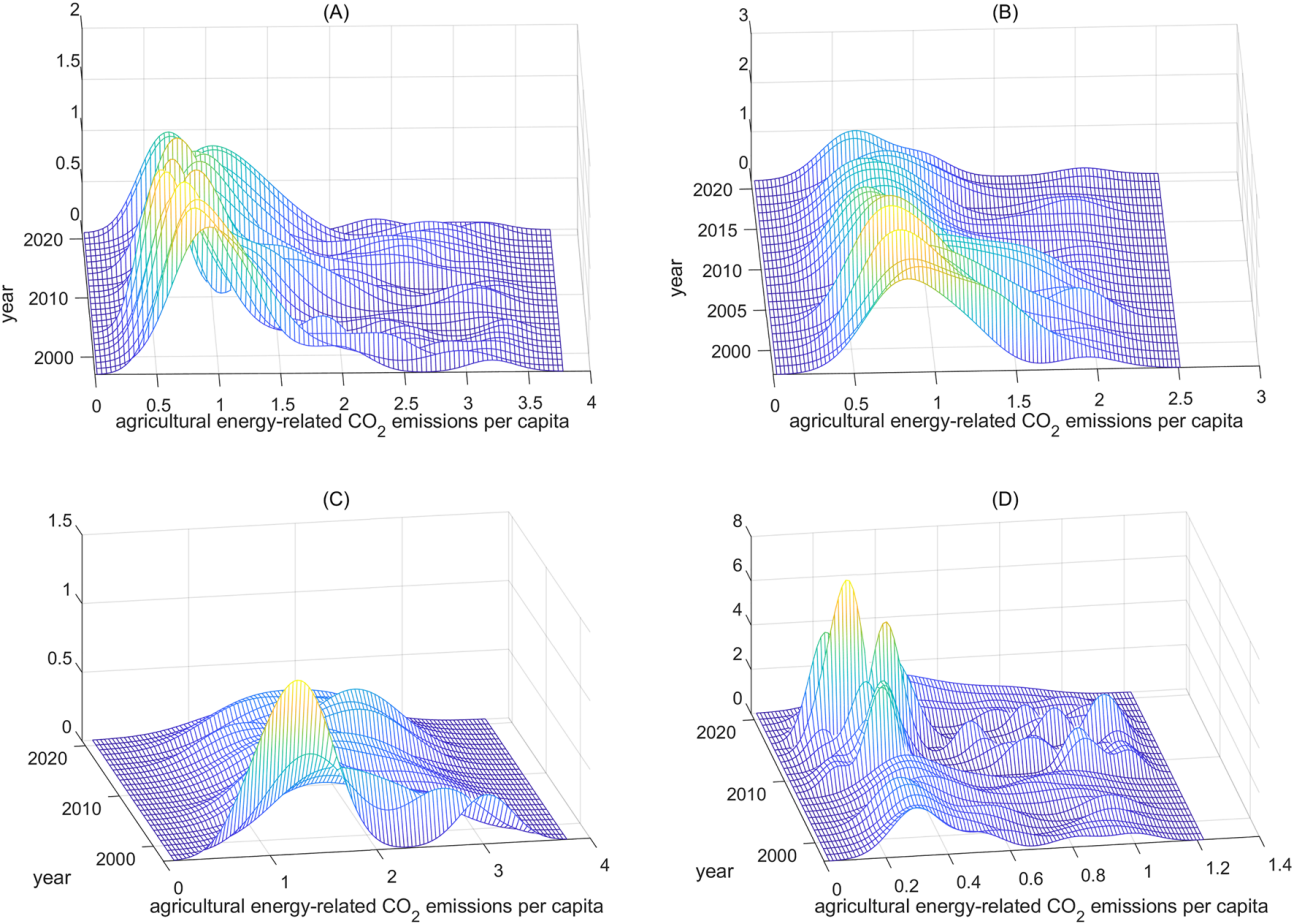
Kernel density curves of agricultural energy-related CO_2_ emissions per capita.

As shown in Fig. 1, the Gaussian kernel function was used to draw the density curves of agricultural energy-related CO_2_ emissions per capita.

In Fig. 1A, it can be seen that the center of the kernel density curves shifted to the right on the whole, indicating a gradual increase in agricultural energy-related CO_2_ emissions per capita in China. Between 1997 and 2021, agricultural energy-related CO_2_ emissions per capita rose from 0.2268 tons to 0.6802 tons in China. The distribution pattern also showed clear multi-peak characteristics, with the peak height fluctuating between increases, decreases, and increases again. As for the peak width, it showed a pattern of narrowing, expanding, and then narrowing again, suggesting a trend of decreasing, increasing, and then decreasing absolute differences in agricultural energy-related CO_2_ emissions per capita among provinces in China. As time went on, the peak height on the right side increased first and then decreased, indicating a first increase and then a gradual weakening of the polarization of agricultural energy-related CO_2_ emissions per capita in China. Additionally, the kernel density curves showed a right-tailing phenomenon, likely due to certain provinces (such as Heilongjiang, Tianjin, Inner Mongolia Autonomous Region, and Zhejiang) experiencing a significantly higher rate of increase in agricultural energy-related CO_2_ emissions per capita compared to others.

Figure 1B revealed the kernel density curves of agricultural energy-related CO_2_ emissions per capita in major grain-producing area. In the study period, the agricultural energy-related CO_2_ emissions per capita showed an ascending trend, with the emissions per capita reaching 0.3932 tons. It was worth noting that, the agricultural energy-related CO_2_ emissions per capita in Heilongjiang fluctuated between 0.5864 and 0.9087 between 1997 and 2010, then began to rise rapidly, reaching 2.5106 tons in 2021. The center of the kernel density curve exhibited a fluctuating pattern of a right–left–right–left shift from 1997 to 2003. Between 2003 and 2007, there was a significant shift to the right, followed by a brief shift to the left before continuing to move to the right. The center of the kernel density curve shifted to the left during 2017–2019 but then shifted back to the right after 2019. The peak height of the distribution pattern fluctuated within a very small range. The bimodal distribution only appeared in 2000, 2003, 2004, and 2016. This signified that there was no clear polarization in agricultural energy-related CO_2_ emissions per capita in the major grain-producing area. This was due to the strategic policy of “focusing on grain production and promoting coordinated development of agriculture and livestock husbandry” in the major grain-producing area, as well as the gradual alignment of agricultural development models and policies ^16^. The phenomenon of right-tailing in the major grain-producing area was gradually disappearing.

Figure 1C depicted the kernel density curves for per capita agricultural CO_2_ emissions from energy consumption in the major grain-selling area from 1997 to 2021. Throughout the sample period, the per capita agricultural CO_2_ emission from energy consumption in the major grain-selling area was 0.6611 tons. The kernel density curves in Fig. 1C demonstrated a gradual shift to the right, indicating an increase in agricultural energy-related CO_2_ emissions per capita. Additionally, the peak height showed a pattern of decline, rise, decline, rise, and decline. Notably, there was no significant polarization phenomenon or tailing phenomenon.

As demonstrated in Fig. 1D, the per capita CO_2_ emissions from agricultural energy consumption in the grain balanced area was 0.3348 tons over the study period. Overall, the center of the kernel density curve shifted noticeably to the right. Additionally, the peak height experienced a fluctuation of rising-falling-rising-rising-falling. Furthermore, the distribution was bimodal and multi-modal, indicating a significant polarization phenomenon in the grain balanced area. In addition, there has been a noticeable increase in the right tailing phenomenon since 2017. Specially, since 2017, the per capita CO_2_ emissions from agricultural energy consumption in Xinjiang, Shanxi, and Guizhou have been much higher than in other provinces in the region.

Overall, the per capita agricultural CO_2_ emissions from energy consumption in China had an increasing trend. However, the distribution and evolution patterns of the emissions in three grain production functional areas varied significantly. Using only kernel density estimation was insufficient in revealing the level and driving factors of inequality in per capita agricultural energy-related CO_2_ emissions. Thus, further analysis was necessary to understand the inequality and the influencing factors.

### Inequality in per capita agricultural energy-related CO_2_ emissions and its decomposition

Based on the Kaya-Theil approach, this study investigated the effects of three kaya factors, namely the CO_2_ emission intensity, energy intensity, and agricultural value added per capita, on the inequality of per capita agricultural CO_2_ emissions from energy consumption in China and three grain production functional areas.

#### Inequality and decomposition of agricultural energy-related CO_2_ emissions per capita in China

**Figure 2:**
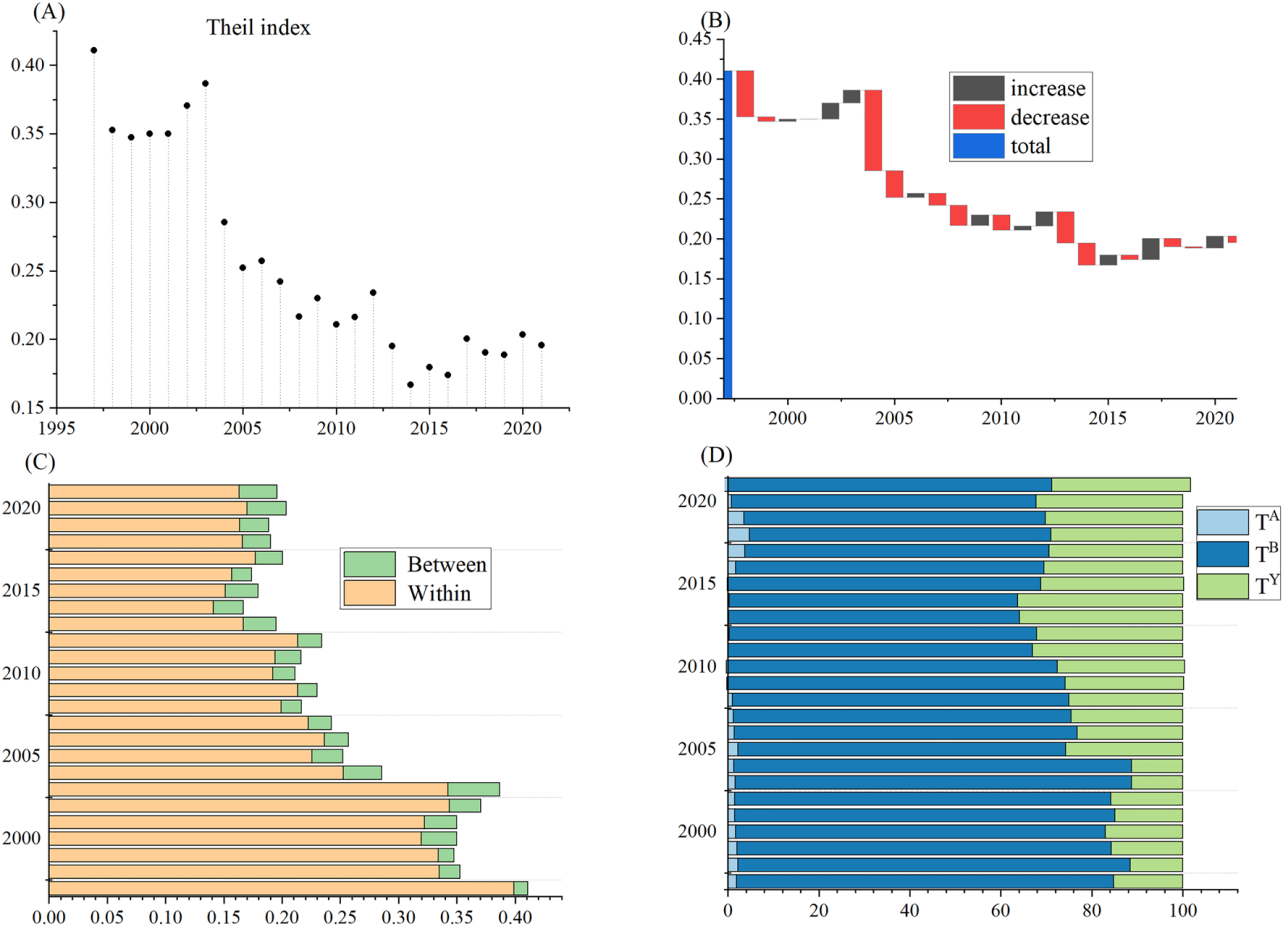
Decomposition of inequality in China’s agricultural energy-related CO_2_ emissions per capita.

Figure 2A revealed that the Theil index of CO_2_ emissions per capita from agricultural energy consumption in China showed a downward trend, with the Theil index falling from 0.4109 in 1997 to 0.1957 in 2021. In the process of decline, there were significant rebounds in 2002, 2003, and 2012 (Fig. 2A). Over the period, 11 years were marked by increases and 13 years were marked by decreases instead. Moreover, the decrease in amplitude was greater than the increase (Fig. 2B). However, the Theil index consistently remained above zero. This indicated that inequality in agricultural CO_2_ emission per capita from energy consumption was still severe in China.

In terms of spatial differences (Fig. 2C), within-group inequality was the major source of the national inequality, significantly greater than between-group inequality when 28 provinces were zoned into three grain production functional areas. That was to say the agricultural energy-related CO_2_ emissions per capita in China exhibited regional characteristics, with significant differences among provinces within the same grain production functional area. Specifically, the within-group inequality has decreased slowly from 0.3991 in 1997 to 0.1634 in 2021, implying a gradual weakening of inequality in agricultural energy-related CO_2_ emissions per capita within a single grain production functional area. However, it was a noteworthy point that the between-group inequality increased from 0.0118 in 1997 to 0.0323 in 2021, indicating a widening gap between grain production functional areas.

With respect to the driving factors (Fig. 2D), energy intensity was found to be the primary factor influencing inequality in agricultural energy-related CO_2_ emissions per capita in China, followed by the agricultural economic development. The contribution of CO_2_ emission intensity was found to be the least. Interestingly, the effect of energy intensity on inequality showed a pattern of first decreasing and then increasing. This outcome implied that the regional gap in agricultural energy efficiency in China was widening, which was similar to the results of previous studies on the spatial differences in agricultural energy efficiency^10^. The result was also similar to the conclusion obtained by the previous literature, which showed that the growth of agricultural energy efficiency in China was sluggish, and its evolution trend gradually tended to spread from relatively concentrated^71^. Therefore, while improving agricultural energy efficiency, it was necessary to further narrow the regional gap. The results further confirmed the conclusions of existing relevant literature^72,73^.

The effect of agricultural energy structure remained relatively stable throughout the study period. This was because China’s agricultural energy consumption has been dominated by coal, diesel and gasoline, which was consistent with previous research^10^. However, the effect of agricultural economic development gradually enhanced, likely due to the differentiation of grain production functional areas, which led to an increase in regional differences in agricultural activity intensity and industrial structure^74^. Overall, the agricultural economic development and energy intensity played positive roles in increasing the Theil index. However, the effect of agricultural energy structure had a negative impact on the Theil index in certain years, such as 2009, 2010, 2015, and 2021. Since 2010, China paid more attention to the improvement of the agricultural energy structure and put forward specific optimization measures and targets. The energy consumption structure of the agricultural sector in China was developing in a more optimized direction^75^. This implied that the adjustment and improvement of energy structure could alleviate the inequality of CO_2_ emissions from agricultural energy consumption to some extent.

#### Inequality and decomposition of agricultural energy-related CO_2_ emissions per capita in major grain-producing area

**Figure 3:**
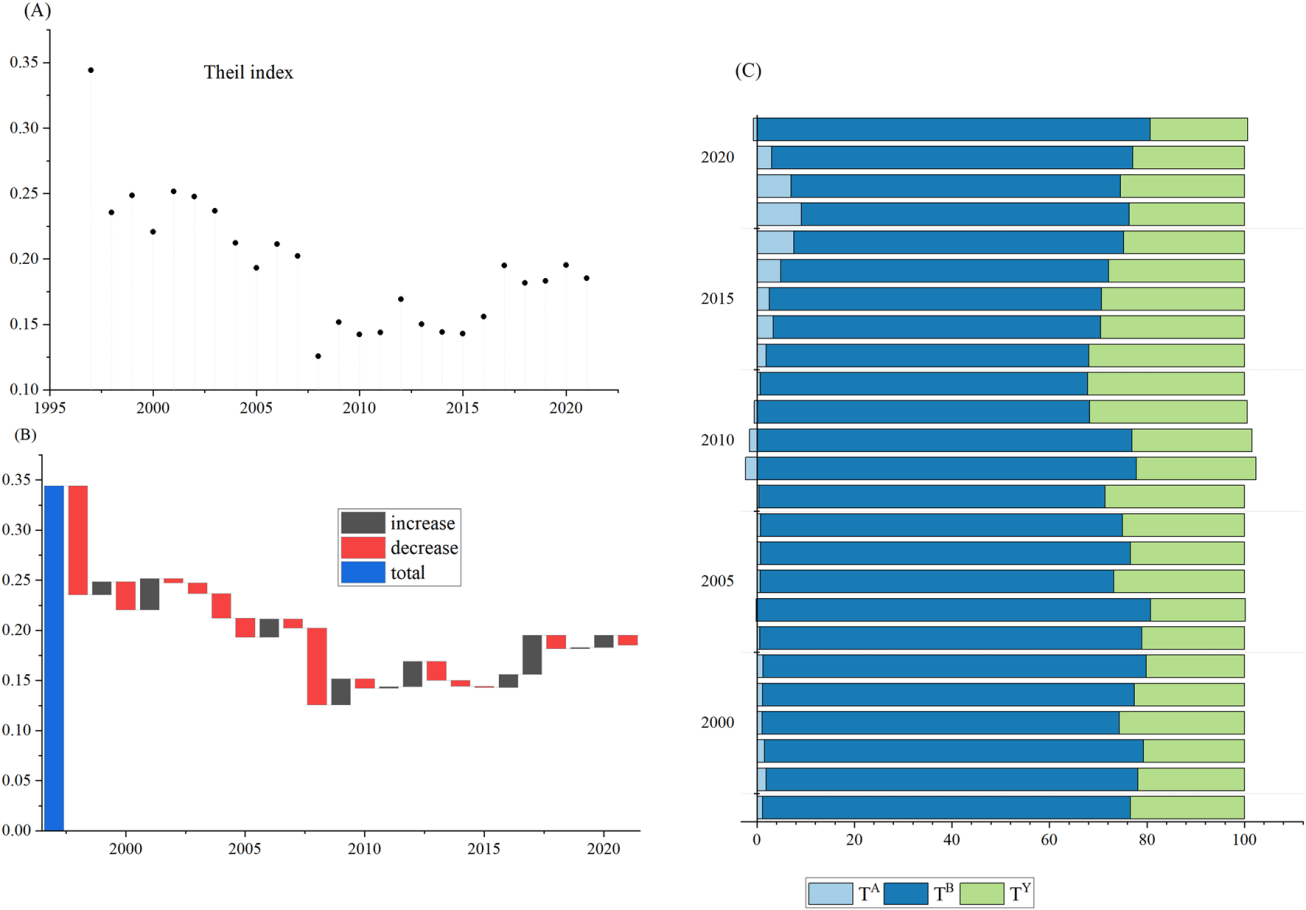
Decomposition analysis of Theil index in the major grain-producing area.

The Theil index of agricultural energy-related CO_2_ emissions per capita in major grain-producing area, as well as the changes and the decomposition results of the Theil index, were all illustrated in Fig. 3.

As shown in Fig. 3A, the Theil index of agricultural energy-related CO_2_ emissions per capita in major grain-producing area took an irregular V-shaped, with a rapid decline before 2008 and a fluctuating rise after 2008 (Fig. 3A). The lowest value of the Theil index reached at 0.1258 in 2008. During the rapid decline stage, there were three years marked by increases and the remaining years marked by decreases (Fig. 3B). Whereas, since 2008, the Theil index only showed decreases in a few years.

As for driving factors, the energy intensity contributed most to the inequality index, followed by the agricultural economic development, while the CO_2_ emission intensity had a minimal effect (Fig. 3C). In major grain-producing area, the main reason for the decrease in the inequality index was the agricultural energy intensity. The contribution of the CO_2_ emission intensity in major grain-producing area increased rapidly from 2011, reaching its peak in 2018 before declining rapidly. It was worth noting that in 2004, 2009–2011, and 2021, the CO_2_ emission intensity had a negative impact on the inequality. When looking at 2011 as a dividing point, the contribution of agricultural economic development experienced two stages of fluctuating rise and rapid decline. The results were probably due to the reforms in agricultural development mode. Since 2004, China has issued a series of Central Document No.1 focusing on the issues of agriculture, rural areas, and farmers. In 2005, the agricultural tax was completely abolished. Along with the continuous increase of financial support for agriculture, China’s agricultural economy has experienced rapid growth. In 2008, China began promoting the transformation of agricultural development from extensive to intensive. Especially in 2010, China listed the transformation of agriculture development mode as the priority of agricultural and rural work^76^. To some extent, the reduction of inter-provincial differences in agricultural economic development helped to alleviate inequality in the major grain-producing area.

#### Inequality and decomposition of agricultural energy-related CO_2_ emissions per capita in major grain-selling area

**Figure 4:**
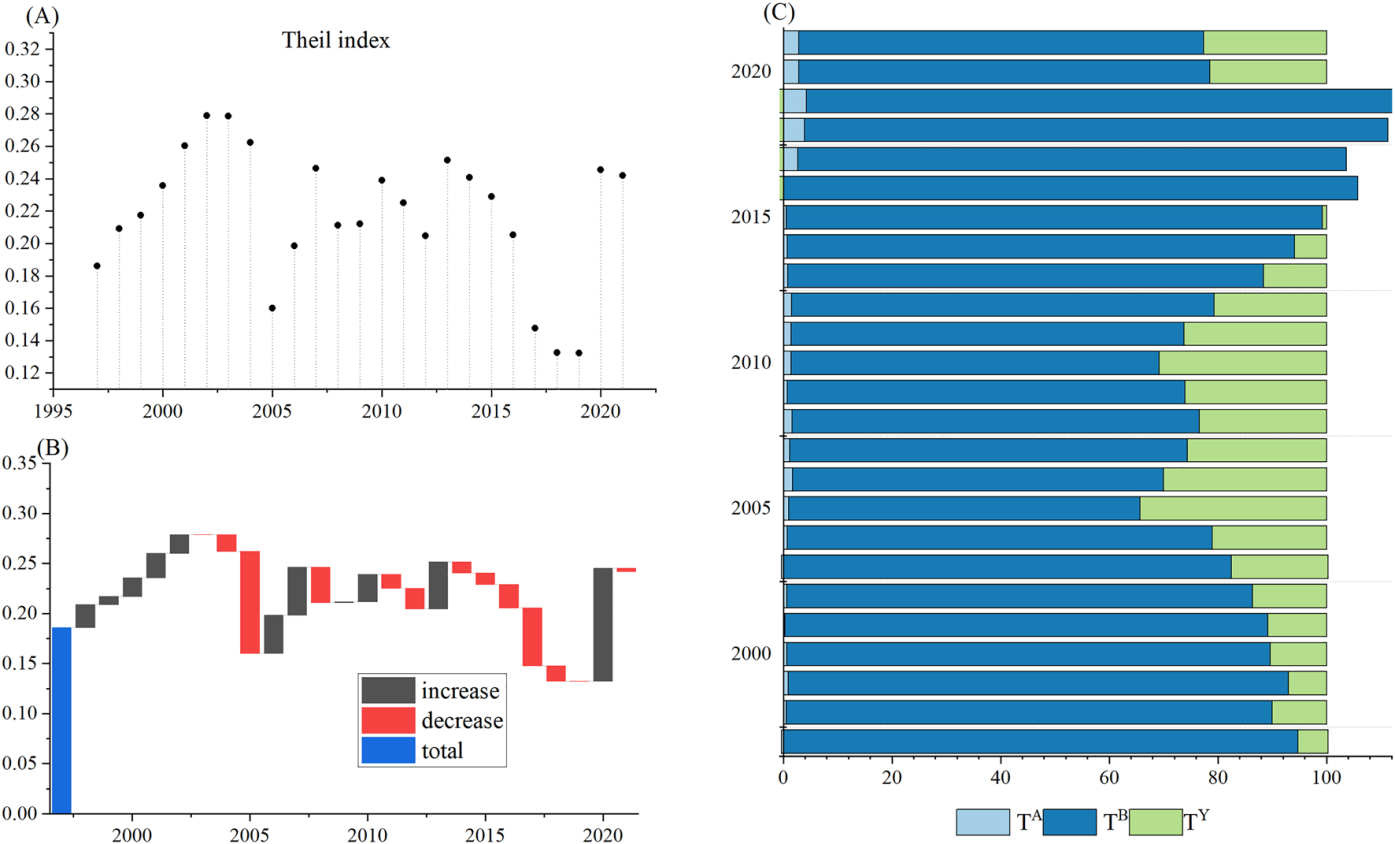
Decomposition analysis of Theil index in the major grain-selling area.

Figure 4 revealed the Theil index of agricultural energy-related CO_2_ emissions per capita in major grain-selling area, the changes in the Theil index and the contribution of three kaya factors.

In general, the Theil index of agricultural energy-related CO_2_ emissions per capita exhibited an M-shaped change in the major grain-selling area (Fig. 4A). Specifically, between 1997 and 2002, the Theil index climbed from 0.1862 to 0.2790. In 2002, the province with the highest agricultural energy-related CO_2_ emissions per capita was Shanghai, which was more than 14 times higher than the lowest province, Fujian. As 85% of Fujian’s land area is mountainous and hilly, the limited availability of cultivated land and the distribution of terraced slope land restricted the use of agricultural energy^77^, resulting in a relatively small amount of agricultural energy-related CO_2_ emissions. However, from 2002 to 2005, the Theil index experienced a period of decline. Then, it was followed by a fluctuating upward trend from 2005 to 2013. Subsequently, from 2013 to 2019, the index once again showed a downward trend. After 2019, the Theil index has increased significantly. That was to say the Theil index fluctuated greatly (Fig. 4B).

In terms of the three kaya factors, the greatest contribution was from energy intensity, followed by agricultural economic development, while the CO_2_ emission intensity contributed the least (Fig. 4C). It was worth noting that from 2016 to 2019, agricultural economic development had a negative impact on the inequality. Only in 1997 and 2003, the CO_2_ emission intensity had a negative impact on inequality.

#### Inequality and decomposition of agricultural energy-related CO_2_ emissions per capita in grain balanced area

**Figure 5:**
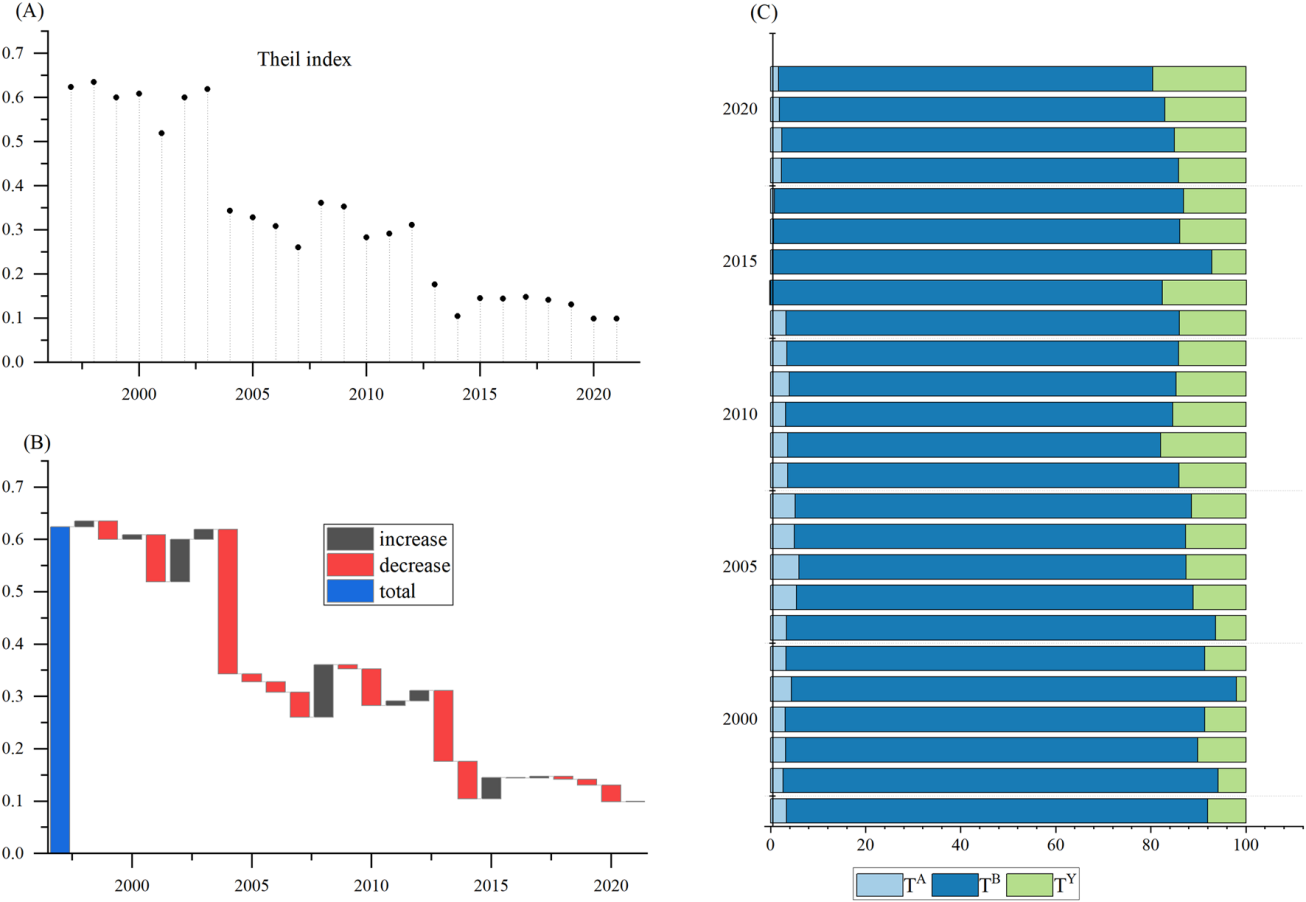
Decomposition analysis of the Theil index in the grain balanced area.

As can be seen from Fig. 5A, the Theil index of agricultural CO_2_ emission per capita from energy consumption exhibited a step-wise decline in the grain balanced area. Between 1997 and 2003, the Theil index fluctuated between 0.6242 and 0.6193. While from 2004 to 2012, it remained relatively stable at around 0.3154. From 2013 to 2021, the Theil index continued to decrease, hovering around 0.1321. Notably, the change stage of the Thiel index was consistent with the stages of China’s agricultural development policy. The provinces in the grain balanced area are located in the central and western regions, and the agricultural production mode and their energy dependence tend to be the same. Previous research on the carbon emission reduction maturity of agricultural energy found a similar result that the inter-provincial disparity in the grain balanced area was significantly reduced^18^. Over the study period, the Theil index in the grain balanced areas experienced 11 years of increases, and the rest years decreased (Fig. 5B).

Among the three kaya factors, energy intensity contributed the most to the Theil index in the grain balanced areas, followed by agricultural economic development, and the contribution of CO_2_ emission intensity was the least (Fig. 5C). It was noteworthy that the contribution of agricultural economic development to the Theil index showed an increasing. The contribution of the CO_2_ emission intensity was characterized by three stages of rising-declining-rising changes.

## Conclusions and policy implications

### Conclusions

Zoning control is a crucial starting point for promoting low-carbon development in China’s agricultural sector. Different functional positioning usually leads to differences in agricultural development modes, which in turn result in different CO_2_ emissions statuses. China’s agricultural energy-related CO_2_ emissions exhibited significant regional disparity in the three grain production functional areas. Thus, it is essential to address the inequality in CO_2_ emissions from agricultural energy consumption in China, especially as the overall CO_2_ emissions were significantly reduced. Assessing the inequality helps promote the coordinated development of agricultural energy-related CO_2_ emission reduction.

By applying the Kernel density estimation, this study focused on the distribution patterns and evolution characteristics of agricultural energy-related CO_2_ emissions per capita in 28 provinces of Mainland China. The Kaya–Theil approach was utilized to assess the inequality in agricultural energy-related CO_2_ emissions per capita among different grain production functional areas and identify the driving factors. The main conclusions drawn from this study were as follows:

1. Overall, China’s agricultural energy-related CO_2_ emissions per capita have been increasing from 0.2268 tons in 1997 to 0.6802 tons in 2021, indicating a growing need for reduction efforts. Additionally, the kernel density estimation revealed an obvious spatial imbalance in agricultural energy-related CO_2_ emissions per capita, with a strong presence of multi-polarization and right-tailing patterns. Specifically, the highest agricultural energy-related CO_2_ emissions per capita was observed in the major grain-selling area, followed by the major grain-producing area, while the grain balanced area had the lowest per capita emissions. Compared to the grain balanced area, which showed clear multi-polarization and right-trailing phenomena, the major grain-producing area and major grain-selling area had weaker polarization and tailing phenomena.
2. The Theil index of agricultural energy-related CO_2_ emissions per capita in China decreased from 0.4109 in 1997 to 0.1957 in 2021. Although there has been a decrease in the inequality in agricultural energy-related CO_2_ emissions per capita in China, it was still relatively high. Upon closer examination, it was evident that the grain balanced area had the highest level of inequality, with a stepwise downward trend. In the major grain-selling area, the inequality followed an M-shaped change. The major grain-producing area, on the other hand, had the lightest degree of inequality and exhibited a V-shaped change. The overall spatial imbalance was mainly due to the within-group inequality, while the contribution of the between-group inequality was relatively low.
3. The decomposition of inequalities indicated that energy intensity was the primary factor influencing the inequality, followed by the agricultural economic development level, while the agricultural energy structure effect was the smallest. This conclusion was valid at both the national and regional levels. Specific to the grain production functional areas, energy intensity has the greatest impact on the inequality in the grain balanced area, followed by the major grain-selling area and the major grain-producing area. Meanwhile, the CO_2_ emission intensity from agricultural energy consumption had negative impact on inequality in certain years. However, the agricultural economic development level only had negative effect on the inequality in the major grain-selling area.

### Policy implications

Based on the above conclusions, some policy implications were put forward as follows:

1. The agricultural energy-related CO_2_ emissions per capita in China had obvious spatial inequality. From the perspective of per capita emissions, the major grain-selling area had the greatest pressure in CO_2_ emission reduction, followed by the major grain-producing area, while the grain balanced area had the least pressure. Therefore, differentiated CO_2_ emission reduction policies should be formulated according to local conditions for different grain production functional areas.
2. To alleviate the overall inequality, it should focus on the reduction of the differences between provinces within grain production functional areas, especially in grain balanced area and major grain-selling area.
3. Agricultural energy intensity was the main factor driving the inequality of agricultural energy-related CO_2_ emissions per capita in China. To reduce this inequality, it is important to promote the continuous improvement of agricultural energy utilization efficiency, particularly in the grain balanced area. However, China’s agricultural energy utilization efficiency is currently low due to insufficient innovation in the agricultural machinery industry. Therefore, it is necessary to increase investment in agricultural machinery research and development, with a focus on developing new technologies that are both energy-efficient and high-performing. Historically, China’s agricultural energy consumption has been dominated by coal, diesel and gasoline. To address this, there is a need to adjust the structure of agricultural energy consumption and accelerate the transition to new energy sources. Additionally, it is important to consider the strategic roles of different grain production functional areas in ensuring China’s food security and to work towards narrowing the development gap between regions and promoting coordinated development in these areas.

### Limitations and future recommendations

This paper proposed some limitations and corresponding recommendations for future research. In this study, the inequality in agricultural energy-related CO_2_ emissions and three potential driving factors were examined thoroughly in China. It must be pointed out that inequality in provincial China was investigated, while the city-level and county-level inequalities were not included due to the availability of data. We must acknowledge that insufficiently detailed geographical scales may weaken the applicability of the findings obtained in this paper. Hence, in future, using more up-to-date data on city and county scales would enhance the accuracy and relevance of the findings. Although this paper combed meticulously relevant literature and strictly screened the driving factors, there may still be some factors not incorporated in the Kaya-Theil model, such as agricultural production structure. Hence, in future, other factors should be further considered to provide more valuable insights.

## Data Availability

The data used and analyzed during the current study are available from the corresponding author upon reasonable request.
